# The Effect of Molecular Isomerism on the Barrier Properties of Polyimides: Perspectives from Experiments and Simulations

**DOI:** 10.3390/polym13111749

**Published:** 2021-05-27

**Authors:** Yiwu Liu, Fengyun Xie, Jie Huang, Jinghua Tan, Chengliang Chen, Linbing Jiang, Wei Sun, Hailiang Zhang

**Affiliations:** 1 National and Local Joint Engineering Center of Advanced Packaging Materials R & D Technology, Key Laboratory of Advanced Packaging Materials and Technology of Hunan Province, School of Packaging and Materials Engineering, Hunan University of Technology, Zhuzhou 412007, China; liuyiwu@hut.edu.cn (Y.L.); xie15273335456@163.com (F.X.); huangjie3@sjtu.edu.cn (J.H.); chenchengliangc@163.com (C.C.); jianglinbing123@163.com (L.J.); s2825948402@163.com (W.S.); 2 Key Laboratory of Polymeric Materials and Application Technology of Hunan Province, Key Laboratory of Advanced Functional Polymer Materials of Colleges, Universities of Hunan Province, College of Chemistry, Xiangtan University, Xiangtan 411105, China; hailiangzhang@xtu.edu.cn

**Keywords:** polyimide, isomerism, structure-property relationship, molecular simulations, barrier properties

## Abstract

A novel carbazole-containing diamine (M-2,7-CPDA) isomer of our previously reported diamine 2,7-CPDA, has been synthesized using a two-step synthesis. Compared with 2,7-CPDA, the substituted position of amino is changed from *para* to *meta* for M-2,7-CPDA. The two diamines were polymerized with pyromellitic dianhydride (PMDA) to prepare two isomeric polyimides (M-2,7-CPPI and 2,7-CPPI), respectively. The effects of *para/meta* isomerism on microstructures and gas barrier performances of the two isomeric polyimides were studied by positron annihilation test, X-ray diffraction and molecular simulation. The results display that *meta*-connected M-2,7-CPPI has less ordered chain structure and weaker hydrogen bonding than *para*-connected 2,7-CPPI, which leads to loose chain stacking and thereby increased free volumes of M-2,7-CPPI. The higher free volumes promote the solubility and diffusivity of gas in M-2,7-CPPI. As a result, the *meta*-linked M-2,7-CPPI shows a lower gas barrier than its *para*-linked analog. The work provides guidance for the design and synthesis of high-performance barrier polymers.

## 1. Introduction

Barrier polymeric materials have widespread applications for the packaging of food, medicine, cosmetics, solar cells and flexible electronics display [1,2,3]. During usage, the barrier polymers can prevent oxygen, water vapor and other gases from penetrating into the package, thus extending the lifetime of products. Nowadays, with the rapid development of flexible electronic devices, there is an increasing demand for flexible polymer substrates with outstanding barriers as well as high heat resistance [4,5,6]. For instance, in the manufacturing of solar cells and active matrix organic light-emitting display devices (AMOLED), the polymer substrates are required to withstand a temperature higher than 400 °C [7,8,9]. At present, the most used barrier polymers include poly(ethylene terephthalate) (PET), polyethylene naphthalate (PEN), poly(vinylidene chloride) (PVDC), polyamide (PA), ethylene-vinyl alcohol copolymers (EVOH) and so on. However, these conventional barrier polymers have low thermostability and are usually used below 200 °C [10,11,12,13,14], which makes their application in flexible substrates difficult.

Aromatic polyimides (PIs) possess exceptional heat resistance, good mechanical and chemical performances, and have become an important material for flexible device substrates [15,16]. Most PIs exhibit a glass transition temperature (*T_g_*) higher than 350 °C. However, conventional PIs possess poor barrier performances, which make it difficult for them to meet the packaging requirements of electronic products [17]. Hence, the research on the barrier performances of PIs is of great significance for promoting their application in flexible substrates.

Polyimides have the advantage of structural diversity. In addition, their molecular structures possess high designability. The performances of PIs can be adjusted by structure modification. Because the substituted positions or arrangement orders are changed, PIs derived from isomeric monomers display different performances [18,19]. Many works have studied the performance of PIs derived from various isomeric diamines or dianhydrides, such as diaminodiphenyl sulfone (DDS) [20], oxydianiline (ODA) [21], bis(aminophenyl) hexafluoropropane [22], biphenyltetracarboxylic dianhydride (BPDA) [23], diphenylsulfonetetracarboxylic dianhydride (DSDA) [24] and diphenylthioether dianhydride (TDPA) [25]. These studies mostly focused on the influence of structural isomerism on the thermal and gas separation performances of PIs. However, no previous works have been conducted to research the isomeric effect on the barrier properties of PIs.

**Figure 1:**
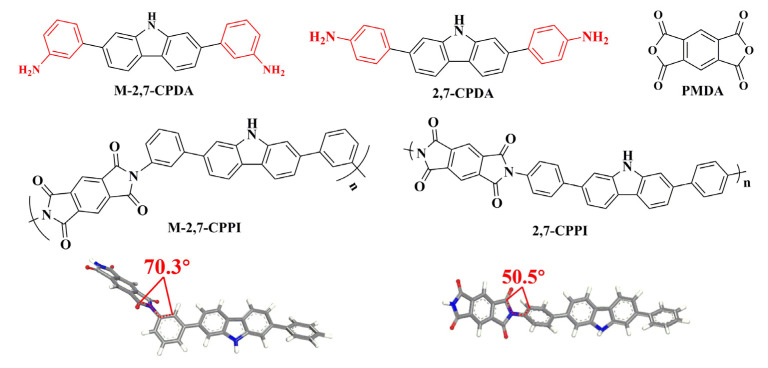
The chemical structures of monomers and the lowest energy conformation of M-2,7-CPPI and 2,7-CPPI repeat units.

In our previous study, a carbazole-containing diamine monomer (2,7-CPDA, see Figure 1) was prepared and reacted with PMDA to obtain a high-barrier polyimide (2,7-CPPI) [26]. Herein, to study the isomeric effect on the barrier performances of PIs, a new diamine (M-2,7-CPDA, see Figure 1) was designed and synthesized. M-2,7-CPDA is an isomer of 2,7-CPDA, in which the substituted position of amino is changed from *para* to *meta*. The M-2,7-CPDA was then reacted with PMDA to prepare polyimide film (M-2,7-CPPI). Figure 1 shows the equilibrated conformations of repeat units for M-2,7-CPPI and 2,7-CPPI. Compared with 2,7-CPPI, M-2,7-CPPI has a larger dihedral angle between imide ring and benzene, thus leading to a more bent and distorted structure. These structural differences will affect the microstructure and thereby the barrier performances of PIs.

Here, the thermal, mechanical and barrier performances of M-2,7-CPPI were investigated and compared with those of 2,7-CPPI. Molecular simulation has become an important means to study the microstructure and gas penetration of polymers at a microscale level [27]. For revealing the effect of structural isomerism on the barrier performances of PIs, molecular simulation, positron annihilation test and X-ray diffraction were adopted to analyze the chain morphology and stacking, free volume, hydrogen bonding, permeation trajectory, diffusion and adsorption behavior of PIs. This helps to understand the relationships between structures and barrier performances and provides theoretical guidance for the development of high-barrier polymers.

## 2. Experimental Section

### 2.1. Experimental Materials

Experimental materials are displayed in Supplementary Materials.

### 2.2. Instrumentation

Characterization instruments are shown in Supplementary Materials.

### 2.3. Molecular Simulation

PIs models with 5 molecular chains were constructed, where each molecular chain contains 25 repeat units. The equilibrated models were used to analyze the free volume feature, aggregation structure, gases solubility and diffusion. The model creation and simulation details are given in the Supplementary Materials.

### 2.4. Synthesis of 2,7-Bis(3-nitrophenyl)-9H-carbazole (M-2,7-CPDN)

3-Nitrophenylboronic acid (75 mmol), 2,7-dibromo-9H-carbazole (30 mmol) and THF (400 mL) were added in a 1000 mL three-necked flask. Then, Aliquat 336 (15 drops) and 2 M aqueous K_2_CO_3_ solution (112.5 mL) were placed and stirred for 0.5 h under argon at room temperature. After that, (Pd [(P(C_6_H_5_)_3_]_4_) was added with stirring for 24 h at 75 °C. The resulting product was chromatographed on a column chromatography (dichloromethane/*n*-hexane (*v/v* = 1/1)). Yield: 85%. IR (KBr, *v*, cm^−1^): 1520 (−NO_2_ stretching), 1262 (C−N stretching), 1086~802 (*δ* Ar−H). ^1^H NMR (400 MHz, DMSO-*d_6_*) δ 11.54 (s, 1H), 8.54 (t, *J* = 2.0 Hz, 2H), 8.38–8.18 (m, 6H), 7.91 (d, *J* = 1.2 Hz, 2H), 7.81 (t, *J* = 8.0 Hz, 2H), 7.62 (dd, *J* = 8.2, 1.6 Hz, 2H); ^13^C NMR (100 MHz, DMSO-*d_6_*) δ 148.95, 143.15, 141.52, 136.19, 134.04, 130.98, 122.73, 122.30, 121.79, 118.71, 110.02; EIMS *m*/*z* (%): 409 (100) [M]^+^, calcd for C_24_H_15_N_3_O_4_, 409.1, Anal. Calcd for C_24_H_15_N_3_O_4_: C 70.41, H 3.69, N 10.26; found: C 70.29, H 3.72, N 10.31.

### 2.5. Synthesis of 3,3′-(9H-Carbazole-2,7-diyl)diamino (M-2,7-CPDA)

Ethanol (450 mL) and M-2,7-CPDN (20 mmol) were poured into a three-necked flask. After heating to 80 °C, 0.5 g of 10% Pd/C catalyst and 16 mL of hydrazine monohydrate were placed into the mixture and stirred for 24 h. Then, the catalyst was cleared away by filtration. The product was collected by recrystallization. Yield: 93%. IR (KBr, *v*, cm^−1^): 3412 (N−H stretching), 1603 (δ N−H), 1262 (C−N stretching), 1086~802 (Ar−H stretching). ^1^H NMR (400 MHz, DMSO-*d*_6_, *δ*): 11.32 (s, 1H, NH), 8.13 (d, *J* = 8.1 Hz, 2H, Ar H), 7.64 (s, 2H, Ar H), 7.38 (dd, *J* = 8.2, 1.0 Hz, 2H, Ar H), 7.14 (t, *J* = 7.8 Hz, 2H, Ar H), 6.97 (s, 2H, Ar H), 6.89 (d, *J* = 7.6 Hz, 2H, Ar H), 6.59 (d, *J* = 7.9 Hz, 2H, Ar H), 5.18 (s, 4H, NH_2_); ^13^C NMR (100 MHz, DMSO-*d*_6_, *δ*): 149.10, 141.87, 140.80, 138.75, 129.39, 121.43, 120.36, 117.86, 114.71, 112.88, 112.48, 108.53; EIMS *m*/*z* (%): calcd for C_24_H_19_N_3_, 349.16; Found: 349. [M]^+^, Anal. Calcd for C_24_H_19_N_3_: C 82.49, H 5.48, N 12.03; found: C 82.17, H 5.66, N 12.49.

### 2.6. Synthesis of Polyimide M-2,7-CPPI

A solution of M-2,7-CPDA (12 mmol) in 18 mL of anhydrous DMF was added in a flask under argon. Then, PMDA (12 mmol) was added. After stirring for 6 h, a viscous poly(amic acid) (PAA) solution was formed. After that, the PAA precursor was casted onto a glass substrate and thermally imidized at 100 °C, 200 °C, 300 °C, and 400 °C for 1 h, respectively. The obtained polyimide membrane (M-2,7-CPPI) was then released from the substrates. IR (KBr, *v*, cm^−1^): 1776 and 1710 (imide carbonyl stretching), 1367 (imide −C−N).

## 3. Results and Discussion

### 3.1. Synthesis and Characterizations of Monomers

**Scheme 1:**
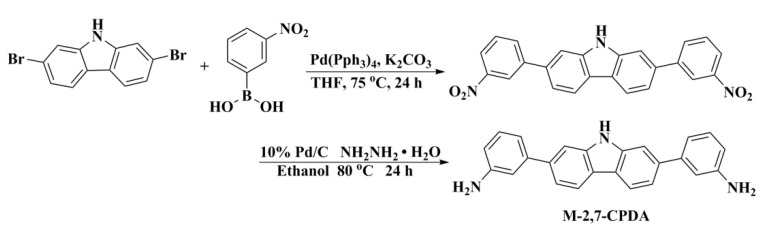
Synthesis route of M-2,7-CPDA.

**Figure 2:**
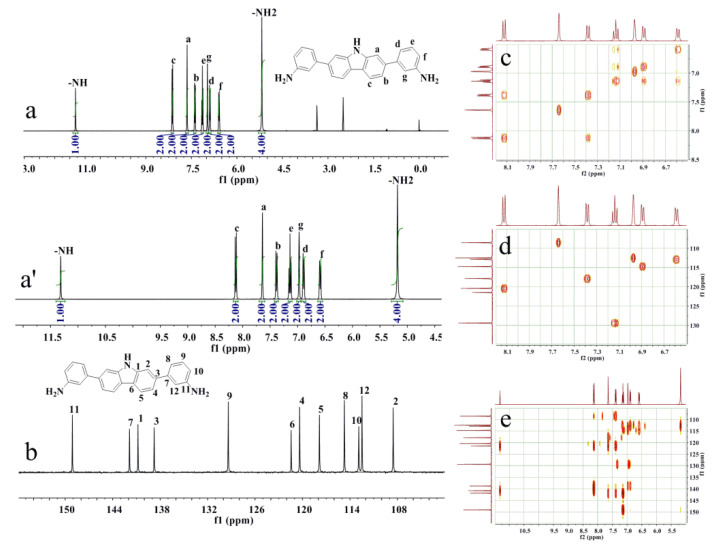
^1^H NMR in the range of 0~13 (**a**), ^1^H NMR in the range of 4.5~11.5 (**a’**), ^13^C NMR (**b**), H-H COSY (**c**), C-H QC (**d**), and C-H BC (**e**) spectra of M-2,7-CPDA in DMSO-*d*_6_.

The synthesis approach of M-2,7-CPDA is shown in Scheme 1. Firstly, M-2,7-CPDN was synthesized from 2,7-dibromo-9H-carbazole and 3-nitrophenylboronic acid by Suzuki reaction. Then, M-2,7-CPDN was reduced to diamine M-2,7-CPDA. NMR, FT-IR, elemental analyses and mass spectra were used to confirm the chemical structure of monomers. Figure S4 and Figure 2 present the NMR spectra of M-2,7-CPDN and M-2,7-CPDA, respectively. Figures S5 and S6 display the mass spectra of M-2,7-CPDN and M-2,7-CPDA, respectively. The FT-IR spectra of M-2,7-CPDN and M-2,7-CPDA are shown in Figure S7. All the above results are in accordance with the pre-designed structures of M-2,7-CPDA and M-2,7-CPDN, certifying the successful synthesis of the M-2,7-CPDA monomer.

### 3.2. Synthesis and Characterizations of PI

**Scheme 2:**
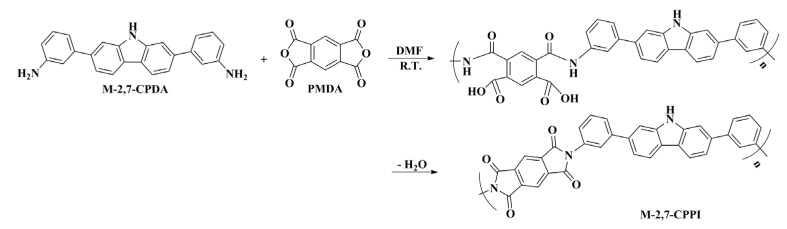
Synthesis route of polyimide.

The polyimide was formed by the two-step polycondensation reaction (Scheme 2). M-2,7-CPDA was polymerized with PMDA to prepare PAA, which was thermally cyclodehydrated to yield M-2,7-CPPI. The weight-average molecular weight (M_w_) of poly(amic acid) was 6.71 × 10^4^ and the polydispersity (PDI) was 1.95. The FT-IR spectra are displayed in Figure S7. The M-2,7-CPDA exhibited absorption bands at 3412 cm^−1^ (N−H stretching) and 1603 cm^−1^ (δ N−H), which are disappeared in M-2,7-CPPI. In addition, M-2,7-CPPI displayed absorption bands at 1776 and 1710 cm^−1^ (carbonyl stretching) and 1367 cm^−1^ (C−N stretching), indicating the successful preparation of M-2,7-CPPI membranes.

### 3.3. Thermal and Mechanical Performances

The thermal performances of M-2,7-CPPI are evaluated by DSC, TGA, TMA and DMA and compared with that of our previously reported *para*-isomer 2,7-CPPI. The results are shown in Figures S8–S11, and related values are summarized in Table 1. M-2,7-CPPI showed superior thermal performances with *T_d5%_* and *T_d10%_* of 563 °C and 601 °C (Table 1), which were comparable to that of *para*-based 2,7-CPPI. The thermostability of the two PIs was better than those of most commercial and reported aromatic PIs. This was mainly because of the planar rigid structures. From TMA and DMA curves, it can be seen that two transitions occur. The one at high temperature corresponded to glass transition (*T_g_*), and the one at low temperature (near 120 °C) corresponded to the secondary *β*-relaxation [28,29,30]. The *β*-relaxation is originated from the rotational motions of local moieties [28]. Two coefficient of thermal expansion (CTE) values of M-2,7-CPPI before and after *β*-relaxation were determined from 40–100 °C and 150–300 °C, respectively. These are 6.39 ppm/K and 49.52 ppm/K, whereas *para*-based 2,7-CPPI exhibited a relatively low CTE of 2.89 ppm/K. The thermal expansion property of PI membranes is strongly associated with the structural linearity/rigidity of polymer chains [31]. The bent and distorted structure of M-2,7-CPPI destroyed the chain alignment, thus leading to a high CTE value. The 2,7-CPPI exhibited higher mechanical performances than M-2,7-CPPI. The linear polymer backbone structure of *para*-linked 2,7-CPPI promoted the dense chain packing and formation of hydrogen bonding, which in turn resulted in higher mechanical properties and lower CTE.

### 3.4. Barrier Properties

The gas barrier of M-2,7-CPPI is compared with 2,7-CPPI, as shown in Table 2. The *meta*-isomer M-2,7-CPPI showed favorable barrier performances with water vapor transmission rate (WVTR) and oxygen transmission rate (OTR) of 10.2 g·m^−2^·day^−1^ and 12.1 cm^3^·m^−2^·day^−1^, respectively, which are comparable to that of conventional barrier polymers such as PET and PA [32,33]. The *para*-isomer 2,7-CPPI displayed better barrier properties compared to its *meta*-counterpart M-2,7-CPPI. The barrier performance difference between the two isomeric polyimides will be discussed in the following sections from the aspects of aggregation structure, free volume, gas diffusion and solubility.

### 3.5. Aggregation Structure Analysis

**Figure 3:**
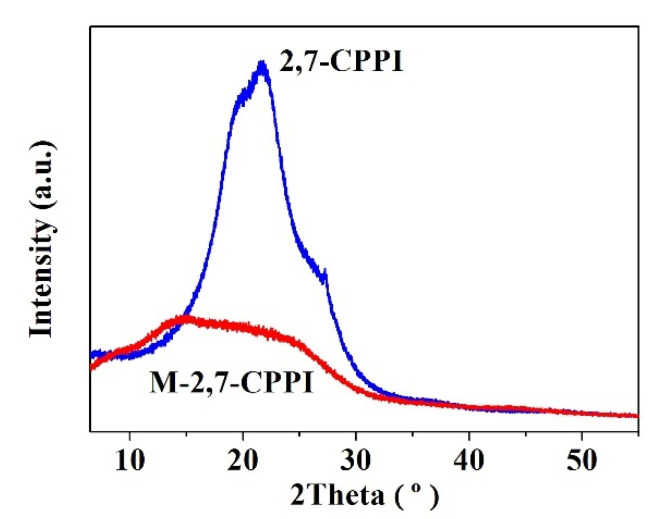
WAXD curve of M-2,7-CPPI and 2,7-CPPI films.

The aggregation structure of PIs was investigated with WAXD, as shown in Figure 3. The *meta*-isomer M-2,7-CPPI showed a diffraction peak at 15.10° with *d*-spacing of 5.86 Å, while in *para*-based 2,7-CPPI, the diffraction peak was shifted to 21.91° with *d*-spacing low to 4.05 Å (Table 3), implying the loose molecular packing of M-2,7-CPPI. The density of M-2,7-CPPI was smaller than that of 2,7-CPPI (Table 3). Additionally, the chain stacking was determined by radial distribution functions (RDFs). The interchain RDFs based on all the C atoms in the benzene ring and all N atoms in the imide ring are analyzed and displayed in Figure S12a,b, respectively. M-2,7-CPPI demonstrated a lower *g(r)* value than 2,7-CPPI in Figure S12a. The chain packing difference between the two isomeric polyimides may be a consequence of chain morphology.

**Figure 4:**
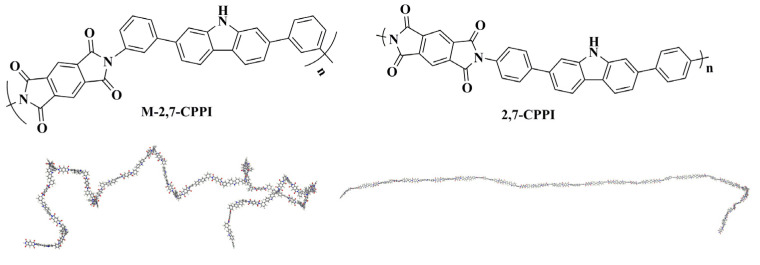
The polymer chain conformations of M-2,7-CPPI and 2,7-CPPI under the lowest energy.

Figure 4 illustrates the polymer chain conformation of M-2,7-CPPI and 2,7-CPPI with 25 repeat units under the lowest energy. The *meta*-isomer M-2,7-CPPI showed bent and distorted chain structure, whereas that of 2,7-CPPI was linear and regular. In addition, the radius of gyration (*R_g_*) was studied for the two isomeric polyimides. The *R_g_* as a function of time for the equilibrated M-2,7-CPPI and 2,7-CPPI are illustrated in Figure S13. The average *R_g_* values are given in Table 3. 2,7-CPPI showed a larger *R_g_* than M-2,7-CPPI, indicating its more stretched chain structure [34].

### 3.6. Hydrogen Bonds Analysis

**Figure 5:**
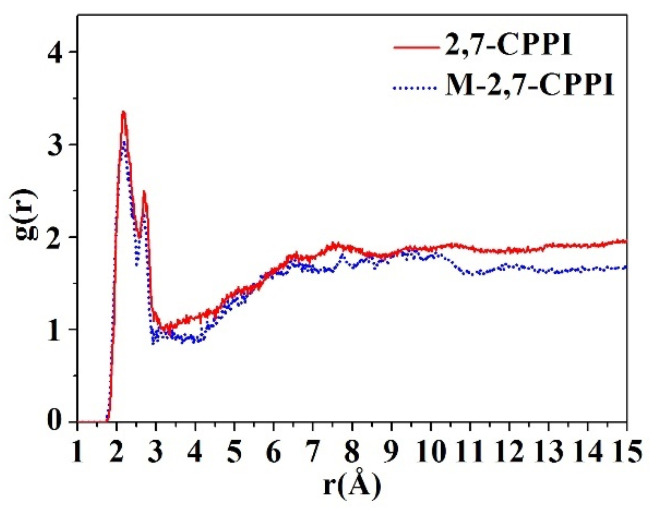
The RDFs of the hydrogen atoms of −HN− in carbazole and oxygen atoms of O=C− in imide rings for M-2,7-CPPI and 2,7-CPPI.

**Figure 6:**
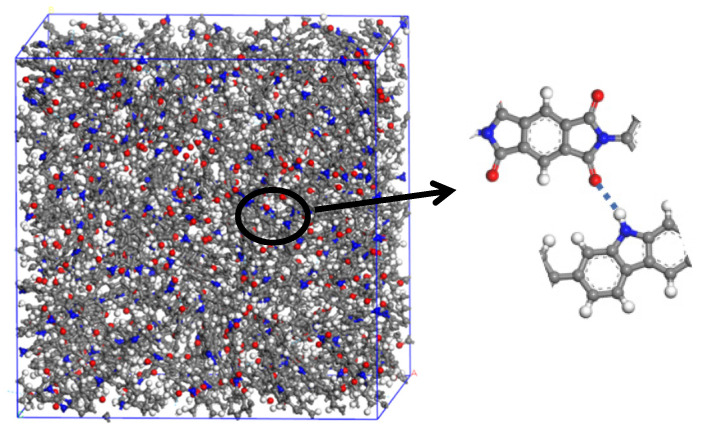
H-bonds between −HN− and O=C− in simulation cell of M-2,7-CPPI.

Intermolecular forces also affect chain stacking. For M-2,7-CPPI and 2,7-CPPI, hydrogen bonds can be formed between O=C− and −NH−. The radial distribution functions (*g_AB_(r)*) of O atoms in O=C− and H atoms in −HN− were determined for M-2,7-CPPI and 2,7-CPPI to study the hydrogen bonds. The result is given in Figure 5. Hydrogen bond forces are considered to have the distances between atoms of 2.6–3.1 Å [35]. In Figure 5, peaks were observed in the range of 2.6–3.1 Å for the two PIs, proving that hydrogen bonds were formed. Obviously, 2,7-CPPI displayed a larger *g*(*r*) value than M-2,7-CPPI in that range, implying there are more hydrogen bonds in the 2,7-CPPI matrix. Figure 6 illustrates the hydrogen bonds generated in the M-2,7-CPPI cell, verifying the presence of hydrogen bonds between O=C− and −NH−. Statistical analysis indicated that 38 and 49 hydrogen bonds were generated in the M-2,7-CPPI and 2,7-CPPI cells, respectively (Table 3). When the linkage was changed from *para* to *meta*, the regularity of polymer chains in M-2,7-CPPI was decreased, and the compact chain stacking was hampered. The less ordered and packed structure of M-2,7-CPPI hindered the formation of hydrogen bonds, leading to a decreased number of hydrogen bonds. This also can be revealed from the cohesive energy density (CED) results in Table 3. The CED of 2,7-CPPI was 570 J/cm^3^, which decreased to 456 J/cm^3^ for M-2,7-CPPI. The distorted chain structure and weak hydrogen bonding gave rise to the loose chain packing of M-2,7-CPPI.

### 3.7. Free Volumes Analysis

#### 3.7.1. Positron Annihilation Test

**Figure 7:**
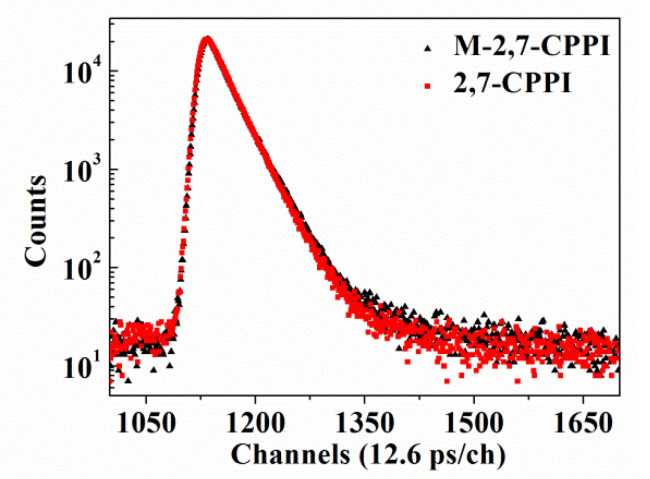
Positron lifetime spectra measured for the M-2,7-CPPI and 2,7-CPPI films.

The molecular chain morphology and aggregation structure have a substantial effect on the free volumes of PIs and thereby the barrier performances [36,37]. Positron annihilation lifetime spectroscopy (PALS) was utilized to analyze the free volumes of PIs. The positron lifetime plots and data are shown in Figure 7 and Table 4. Based on the second lifetime component (*τ*_2_) and its intensity (*I*), the mean radius (*R*) and size (*V_f2_*) of free volume and relative fractional free volume (*FFV*) were determined according to our previous study [26]. The *para*-isomer 2,7-CPPI showed *R* and *FFV* of 2.14 Å and 6.82%, whereas, for *meta*-based M-2,7-CPPI, those were increased to 2.26 Å and 7.46%.

#### 3.7.2. Molecular Simulations

**Figure 8:**
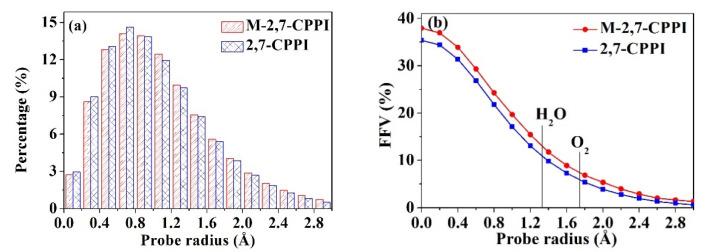
Distributions of void radius (**a**) and fractional free volume (**b**) as a function of probe radius in M-2,7-CPPI and 2,7-CPPI. The kinetic radii of O_2_ and H_2_O are indicated by vertical lines.

Free volume features, including size distribution and connectivity, were considered as key parameters determining gas transport performance [38,39]. Molecular simulations were conducted to study the free volume. The distributions of void size were shown in Figure 8a. Compared with para-isomer 2,7-CPPI, meta-based M-2,7-CPPI had a greater number of holes with a radius larger than 0.8 Å, but fewer holes with a radius smaller than 0.8 Å. The kinetic radii of H_2_O and O_2_ are larger than 0.8 Å (O_2_: 1.73 Å, H_2_O: 1.325 Å). This suggested that M-2,7-CPPI had more cavities for H_2_O and O_2_ permeation in comparison with 2,7-CPPI, which led to high gas permeability and therefore decreased barrier performances of M-2,7-CPPI.

**Figure 9:**
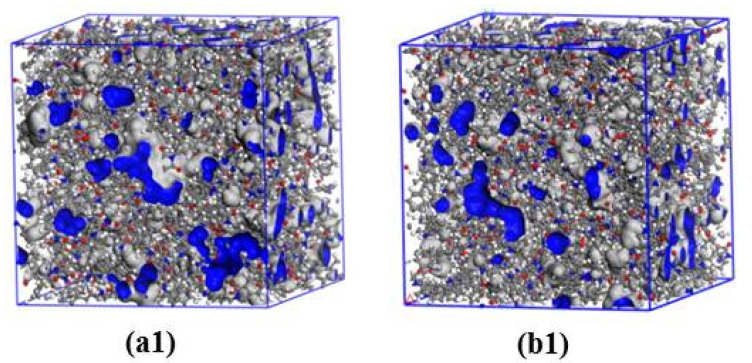

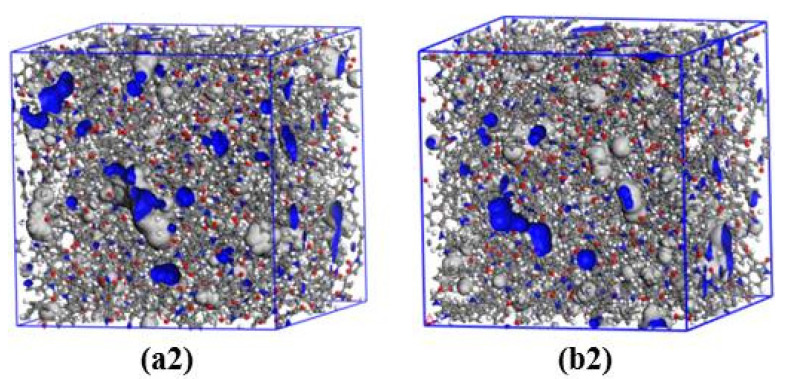
Three-dimensional representation of H_2_O and O_2_ accessible volume for (**a1**,**a2**) M-2,7-CPPI and (**b1**,**b2**) 2,7-CPPI. (grey: Van der Waals surface; blue: Connolly surface; (**a1**,**b1**): probe radius of 1.325 Å; (**a2**,**b2**): probe radius of 1.73 Å).

The relationships between probe radius and *FFV* of the two PIs are studied and shown in Figure 8b. With the increase of probe radius, the *FFV* of the two polyimides decreased. The *meta*-based M-2,7-CPPI exhibited larger FFV than 2,7-CPPI in the whole radius range. The total fractional free volume (*FFV*^0^) was simulated using a probe radius of 0 Å. The *FFV* (O_2_) and *FFV* (H_2_O) were also calculated using the kinetic radii of O_2_ and H_2_O as probe radii. Table 4 lists the *FFV*^0^, *FFV* (O_2_) and *FFV* (H_2_O) values. The *meta*-based M-2,7-CPPI showed higher *FFV* values than 2,7-CPPI, in agreement with the PALS analysis. The accessible volume morphologies of H_2_O and O_2_ in the two PIs are presented in Figure 9. The *para*-isomer 2,7-CPPI possessed smaller and disconnected holes, while those in *meta*-based M-2,7-CPPI were bigger and well-connected. Changing the linkage from *para* to *meta* reduced the molecular chain order, which hindered the tight chain packing and weakened the intermolecular hydrogen bonding. Consequently, the number and size of free volumes increased, thus bringing about high gas permeability and thereby decreased barrier performances of M-2,7-CPPI.

### 3.8. Gas Transport Behavior

From the perspectives of gas diffusion and solubility, gas transport behavior is investigated to understand the barrier performance difference between the two isomeric polyimides.

#### 3.8.1. Gas Diffusivity

**Figure 10:**
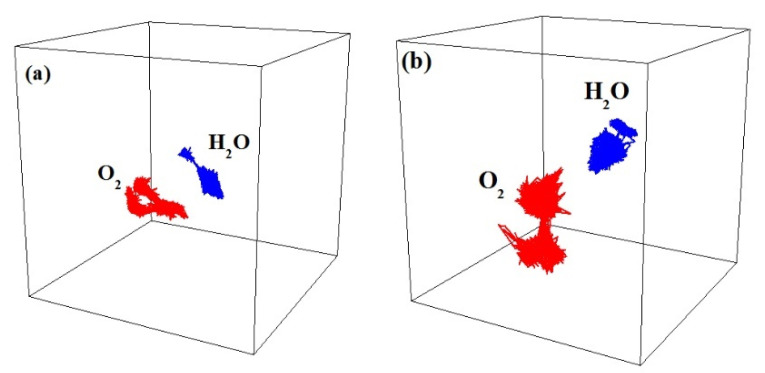
The representative trajectories of O_2_ and H_2_O in (**a**) 2,7-CPPI and (**b**) M-2,7-CPPI.

The diffusivity behaviors, including trajectory and displacement of O_2_ and H_2_O in the PI matrices, were studied and shown in Figure 10 and Figure S14, respectively. Oxygen and water molecules had two motion statuses in the PI matrix. One was the oscillation in the voids, and the other was occasional jumps from one void to an adjacent void [40]. As shown in Figure 10 and Figure S14, the movement distance and frequency of oxygen and water in *para*-isomer 2,7-CPPI were lower, whereas those in *meta*-based M-2,7-CPPI were larger, indicating that oxygen and water had higher mobility in the M-2,7-CPPI matrix.

**Figure 11:**
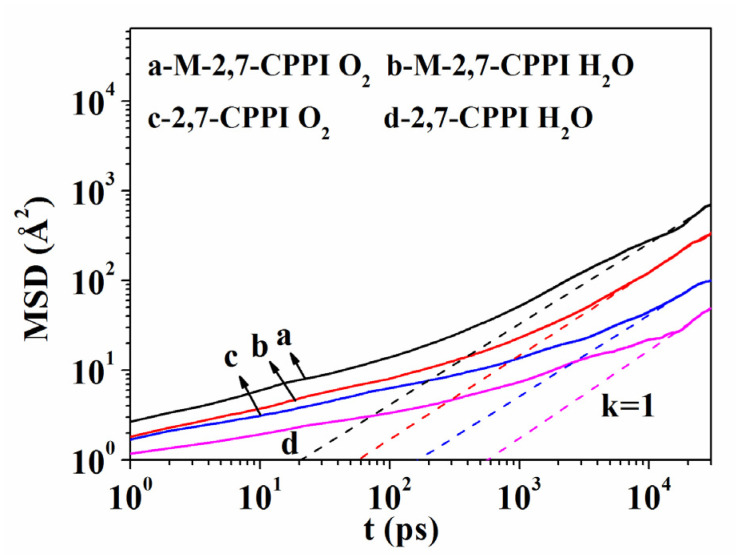
MSD of O_2_ and H_2_O in M-2,7-CPPI and 2,7-CPPI as a function of time.

The MSD vs. time plots of H_2_O and O_2_ in the two polyimides are presented in Figure 11. According to Einstein’s equation, the diffusion coefficients (D) of O_2_ and H_2_O were determined from normal diffusion interval, in which the plot slope was unity. The D values are shown in Table 5. The *meta*-based M-2,7-CPPI displayed larger D values of H_2_O and O_2_ compared with those of 2,7-CPPI. Changing the linkage from *para* to *meta* disrupted chain packing and weakened intermolecular forces, thus leading to increased free volume. As a result, the diffusion coefficients of H_2_O and O_2_ were enhanced in M-2,7-CPPI. Additionally, it can be found that in a fixed PI matrix, the D of O_2_ was larger than that of H_2_O, even though the kinetic radius of O_2_ was bigger. This was mainly owing to the high interaction between the polar water molecule and the PI, impeding H_2_O diffusion [41].

#### 3.8.2. Gas Solubility

**Figure 12:**
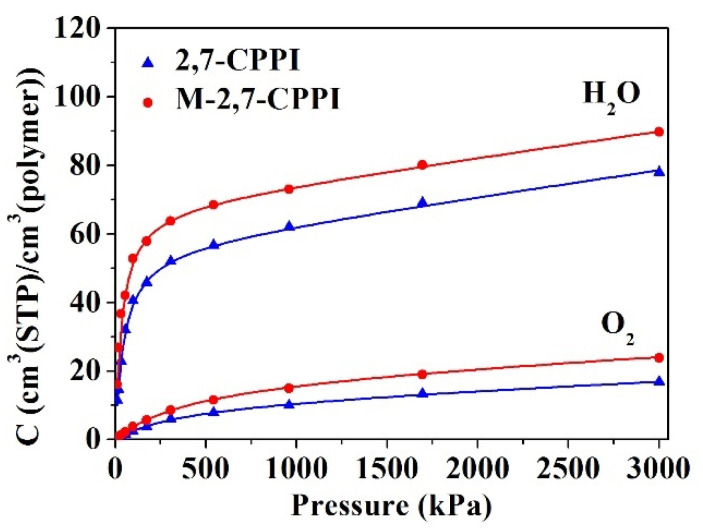
The adsorption isotherms of O_2_ and H_2_O in M-2,7-CPPI and 2,7-CPPI.

**Figure 13:**
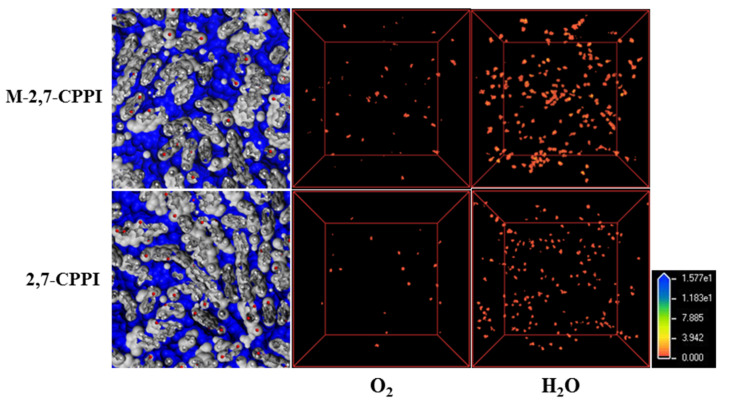
Sorption sites of O_2_ and H_2_O in M-2,7-CPPI and 2,7-CPPI. (Red, yellow, and blue stand for low, medium, and high adsorption amounts, respectively.).

For a better understanding of gas sorption behaviors, the gas sorption isotherms of H_2_O and O_2_ were analyzed and exhibited in Figure 12. The sorption isotherms of H_2_O and O_2_ in the two PIs can be elucidated by a typical dual-mode sorption model [42]. When the pressure was low, the Langmuir’s sorption dominated, which occurred in the micro-voids. At high pressures, Henry mode sorption took place in free volume among the chains. The obtained solubility coefficients (S) in M-2,7-CPPI and 2,7-CPPI are listed in Table 5. The *meta*-based M-2,7-CPPI presented higher S values of H_2_O and O_2_ than *para*-based 2,7-CPPI. In addition, the sorption sites of H_2_O and O_2_ in the two PIs are displayed in Figure 13. M-2,7-CPPI had larger sorption loading of H_2_O and O_2_ than 2,7-CPPI. As discussed above, *meta*-based M-2,7-CPPI possessed a larger number and size of free volumes, providing more sites for gas sorption. This was the main reason for the higher S value in M-2,7-CPPI. It is worth mentioning that the S of H_2_O was higher than that of O_2_ in a fixed PI. This was mostly ascribed to the high affinity between PI and H_2_O and the smaller size and high critical temperature of H_2_O [43].

#### 3.8.3. Gas Permeability

The permeability coefficient (P) was calculated with the equation P = D × S as elucidated by the solution-diffusion mechanism [44] and is shown in Table 5. The simulated P of O_2_ and H_2_O in the two isomeric polyimides displayed the same variation trend with the experimental values, proving the viability of molecular simulation on gas permeability. Altering the linkage from *para* to *meta* impeded the dense chain stacking and thus increased free volumes, promoting the gases diffusivity and solubility. Consequently, the *meta*-based isomer M-2,7-CPPI showed increased gas permeability and therefore reduced barrier properties.

## 4. Conclusions

A diamine (M-2,7-CPDA) bearing carbazole moiety was synthesized by a two-step method. This diamine is an isomer of our previously reported diamine 2,7-CPDA, in which the substituted position of amino is altered from *para* to *meta*. Both diamines were polymerized with PMDA to obtain two isomeric polyimides (M-2,7-CPPI and 2,7-CPPI). The two isomeric PIs showed comparable thermal stability. *Meta*-linked M-2,7-CPPI had lower mechanical properties and higher CTE than *para*-linked 2,7-CPPI. Molecular simulation, PALS and WAXD were employed to explore the effect of molecular isomerism on barrier performances of polyimides. Compared with *para*-linked 2,7-CPPI, *meta*-linked M-2,7-CPPI displayed loose chain stacking caused by the less ordered chain structure and weaker hydrogen bonding, which led to higher *FFV* of M-2,7-CPPI. The higher free volumes benefited the gases diffusivity and solubility in M-2,7-CPPI matrix. Consequently, the *meta*-linked M-2,7-CPPI displayed a lower gas barrier than its *para*-linked analog. This research provides inspiration for the design of high-performance barrier polymers.

## Figures and Tables

**Table 1:** Thermal and mechanical properties of M-2,7-CPPI and 2,7-CPPI.

PI	*T_g_*^1^ (°C)	*T_g_*^2^ (°C)	*T_d5%_* (°C)	*T_d10%_* (°C)	CTE ^3^ (ppm∙K^−1^)	Tensile Strength (MPa)	Tensile Modulus (GPa)	Elongation at Break (%)
2,7-CPPI ^4^	413	437	556	580	2.89	143.8 ± 3.5	4.5 ± 0.2	9.3 ± 0.4
M-2,7-CPPI	395	437	563	601	6.39, 49.52	120.0 ± 3.1	2.0 ± 0.2	7.6 ± 0.3

^1^ measured by DSC; ^2^ measured by DMA; ^3^ The two coefficient of thermal expansion (CTE) values of M-2,7-CPPI are determined from 40~100 °C and 150–300 °C, respectively; ^4^ The values are obtained from Ref. [26].

**Table 2:** Barrier properties M-2,7-CPPI and 2,7-CPPI films.

PIs	WVP (g·mil·m^−2^·day^−1^)	OP (cm^3^·mil·m^−2^·day^−1^)	WVTR (g·m^−2^·day^−1^)	OTR (cm^3^·m^−2^·day^−1^)
2,7-CPPI ^1^	0.3 ± 0.02	0.5 ± 0.03	0.1 ± 0.01	0.2 ± 0.01
M-2,7-CPPI	30.3 ± 0.6	35.8 ± 0.6	10.2 ± 0.2	12.1 ± 0.2

^1^ The barrier properties values are obtained from Ref. [26].

**Table 3:** Physical properties of the M-2,7-CPPI and 2,7-CPPI.

PI	Density (g∙cm^−3^)	*2θ* (°)	*d*-Spacing (Å)	*R_g_* (Å)	N ^1^ (H-Bonds)	CED (J∙cm^−3^)
2,7-CPPI	1.57	21.91	4.05	54.3	49	570
M-2,7-CPPI	1.56	15.10	5.86	47.5	38	456

^1^ The number of hydrogen bonds in the simulation cells.

**Table 4:** The analyzed data for the positron lifetime in the M-2,7-CPPI and 2,7-CPPI films.

PI	*τ*_1_ (ns)	*I*_1_ (%)	*τ*_2_ (ns)	*I*_2_ (%)	*R* (Å)	*V_f_*_2_ (Å^3^)	*FFV*^1^ (%)	*FFV*^2^ (O_2_,%)	*FFV*^2^ (H_2_O,%)	*FFV*^0 2^ (%)
2,7-CPPI ^3^	0.17	7.4	0.34	92.4	2.14	41.03	6.82	6.29	11.51	35.33
M-2,7-CPPI	0.16	12.6	0.35	85.7	2.26	48.33	7.46	7.05	12.32	37.90

^1^  *FFV* determined by PALS. ^2^ Free volume parameters calculated by simulations, *FFV* (O_2_), *FFV*(H_2_O) and *FFV*^0^ based on probe radii of 1.73 Å, 1.325 Å and 0 Å, respectively. ^3^ The values are obtained from Ref. [26].

**Table 5:** Simulated diffusion coefficients, solubility coefficients and permeability for O_2_ and H_2_O in M-2,7-CPPI and 2,7-CPPI.

PIs	D ^1^		S ^2^		P ^3^	
	H_2_O	O_2_	H_2_O	O_2_	H_2_O	O_2_
2,7-CPPI	3.24	6.55	0.64	0.013	2.07	0.09
M-2,7-CPPI	7.84	14.20	2.39	0.044	18.74	0.62

^1^ Units of (10^−8^cm^2^/s). ^2^ Units of (cm^3^(STP) cm^−3^·cmHg^−1^). ^3^ Units of (10^−8^cm^2^·cm^3^(STP)·s^−1^·cm^−3^·cmHg^−1^).

## Data Availability

The data presented in this study are available on request from the corresponding author.

## Supplementary Materials

The following are available online at https://www.mdpi.com/article/10.3390/polym13111749/s1. Materials and instrumentation. Details of molecular simulation. ^1^H NMR, ^13^C NMR spectra and mass spectrum of M-2,7-CPDN. Mass spectrum of M-2,7-CPDA. FT-IR spectra of M-2,7-CPDN, M-2,7-CPDA and M-2,7-CPPI. TGA, DSC, DMA and TMA curves of M-2,7-CPPI film. The *g(r)*, *R_g_* and displacement of O_2_ and H_2_O for M-2,7-CPPI and 2,7-CPPI.
