# Supplementary material for: The Effect of Molecular Isomerism on the Barrier Properties of Polyimides: Perspectives from Experiments and Simulations
Source: Polymers (Basel). 2021 May 27;13(11):1749. doi: 10.3390/polym13111749 (PMC8198498; doi:10.3390/polym13111749)
Supplement: Supplementary file 1 [file polymers-13-01749-s001.zip › polymers-1154771-supplementary.pdf]

Supporting Information

The effect of molecular isomerism on the barrier properties of polyimides: perspectives from experiments and simulations

Yiwu Liu[a], Fengyun Xie[a], Jie Huang[a], Jinghua Tan[a*], Chengliang Chen[a], Linbing Jiang[a], Wei Sun[a], Hailiang Zhang[b]

a. National and Local Joint Engineering Center of Advanced Packaging Materials R & D Technology, Key Laboratory of Advanced Packaging Materials and Technology of Hunan Province, School of Packaging and Materials Engineering, Hunan University of Technology, Zhuzhou 412007, P. R. China

b. Key Laboratory of Polymeric Materials and Application Technology of Hunan Province, Key Laboratory of Advanced Functional Polymer Materials of Colleges, Universities of Hunan Province, College of Chemistry, Xiangtan University, Xiangtan 411105, P. R. China

*To whom all correspondence should be addressed.

E-mail: tjh@hut.edu.cn

Contents

1. Materials and instrumentation 3

2. Details of molecular simulation 5

3. Characterization and properties of monomers and polyimides 13

5. Radius of gyration analysis 17

6. Gas diffusion 18

References 19

1. Materials and instrumentation

1.1 Materials

2,7-Dibromo-9H-carbazole, 3-nitrophenylboronic acid, tetrakis(triphenyl-phosphine)palladium, palladium 10 % on carbon (10 % Pd/C), hydrazine monohydrate (NH_2NH_2•H_2O), potassium carbonate, aliquat 336 (tricaprylylmethylammonium chloride), ethanol, were purchased from Alfa-Aesar company. Pyromellitic dianhydride (PMDA) and 4,4′-diaminodiphenyl oxide (ODA) were bought from Alfa-Aesar company and PMDA was vacuum dried at 110 ℃ for 6 h before use. Analytical grade dimethyl formamide (DMF) was purified by distillation under N_2 atmosphere. Tetrahydrofuran (THF) as analytical grade was acquired from National Pharmaceutical Group Chemical Reagent Co., Ltd.

1.2 Instrumentation

Fourier-transform infrared (FT-IR) spectra were obtained on a BRUKER TENSOR 27 FT-IR spectrometer. The monomers were measured by incorporating samples in KBr disks and PI film was measured by attenuated total reflectance mode. Nuclear Magnetic Resonance spectra (NMR) were recorded using a Bruker AVANCE AV 400 spectrometer. The internal reference was tetramethylsilane (TMS). Elemental analysis and Mass spectra were carried out on a CHNS Elemental Analyzer and a Thermo EI mass spectrometer (DSQ II), respectively. Molecular weight of the polyamic acid was measured by gel permeation chromatography analyses on multi-angle laser light scattering (GPC-MALLS) system (Wyatt Technology Corporation). DMF was used as eluant at a flow rate of 1 mL/min and at the temperature of 50 ℃. Wide angle X-ray diffractograms (WAXD) were obtained by a

Rigaku, Ultema III X-ray diffractometer using a Cu Kα radiation. The density of samples was tested with a MIRAGE SD-200L ALFA electronic density balance (Japanese).

Thermogravimetric analyses (TGA) were performed on a TA thermal analyzer (Q50) under N_2 (flowing rate of 40 mL/min) in the temperature range from 40 to 800 ℃ with heating rate of 20 ℃/min. Differential scanning calorimetry (DSC) curves were obtained with a NETZSCH thermal analyzer (DSC 204). The polyimide was measured at a heating rate of 25 ℃ /min from 20 to 400 ℃ under flowing nitrogen. The glass transition temperature (T_g) of polyimide was taken from the second heating trace after rapid cooling from 400 ℃ at a cooling rate of 40 ℃/min. The midpoints of the transitions in the heat capacity were regarded as $T_g s$ values. Dynamic mechanical analysis (DMA) measurements were performed using a TA dynamic mechanical analyzer (DMA Q800) in the tensile mode. The frequency and heating rate were fixed at 1 Hz and 5 ℃/min, respectively. The coefficient of thermal expansion (CTE) of the film was measured using a thermal mechanical analysis (TMA Q400) instrument over a temperature range from 40 to 350 ℃ with a heating rate of 10 ℃/min. The mechanical properties of the PI films were measured with SANS CMT6103 instrument (Shenzhen, China) according to Standard GB/T16421-1996. The samples were cut from 35~50-μm-thick sheet and the size was 100 mm in length and 10 mm in width. The jaw separation was 50 mm. The jaw speed was first set to 2 mm/min, and then changed to 20 mm/min when the elongation reached 1 mm. The oxygen permeability measurements of PI films were performed

using an Oxtran 2/21 ML instrument of MOCON (Minneapolis, MN) at 0 % RH and 23 ℃, in accordance with ASTM-D3985. The moisture vapor permeability was measured via the model PERMATRAN-W® 3/33 of the Mocon Corporation (USA) at 90 % RH and 37.8 ℃ according to ASTM F-1249. The specimen was fixed on an aluminum foil with the film testing area of 5 cm^2. For each polyimide, five samples were measured and the median value was reported.

Positron lifetime measurements were carried out on a fast-fast coincidence system. Two identical samples with dimension of 10 mm × 10 mm × 1.5 mm were sandwiched between a ^{22}Na positron source with intensity of about 7×10^5 Bq. When the ^{22}Na nuclei emits a positron, it also emits a 1.28 MeV γ-ray simultaneously (within a few picoseconds). The positron lifetime is then determined by the time difference between the emission of the birth gamma ray (1.28 MeV) and the annihilation photon. The time resolution of the lifetime spectrometer is about 220 ps in full width at half maximum (FWHM). Totally 4096 channels with a channel width of 12.6 ps were used to collect the positron lifetime spectrum. The PATFIT program was used to decompose the lifetime spectrum into several exponential components after background subtraction and source correction. The source components (360 ps / 16.88 %, 1.18 ns / 0.95 %) were determined using several reference samples such as Si and Al single crystals. The variance of the fit was ~1.1.

2. Details of molecular simulation

2.1 Construction of polymer microstructures

Biovia Materials Studio software was employed to perform simulations in this study using the COMPASS (Condensed-phase Optimized Molecular

Potentials for Atomistic Simulation Studies) forcefield [1,2]. First, a single polymer chain containing 25 repeat units was built followed by geometry optimization. Then, a periodic cell comprising of 5 such polymer chains was constructed and the total energy of the system was minimized using smart minimizing method. Annealing was performed by the NPT (constant number of particles (N), pressure (P) and temperature (T)) dynamics procedure through heating and cooling the system at 1 atm in the temperature range of 300 to 1000 K in steps of 50 K. The total simulation time for the annealing procedure was 30 ns. The atom-based summation method and Ewald summation method were used to calculate the interactions of non-bond, van der Waals and electrostatic forces, respectively. The annealed cell was later put through a stage-wise equilibration procedure. First the cell was heated to 1000 K, and then the temperature was decreased in several stages to 600 K in steps of 100 K. After this, the temperature was decreased in several stages to 400K in steps of 50 K and then decreased to 300 K in steps of 25 K. Each stage included two consecutive NVT (constant number of particles (N), volume (V) and temperature (T)) and NPT runs at 1 atm and a specific temperature. At each specific temperature the polymer system was simulated for 1000 ps. Overall, the total simulation time for the equilibration procedure was 26 ns. The aim of the procedure was to obtain a refined system that would relax at the experimental density of the amorphous polymer at 1 atm and 300 K. Finally, the cell was relaxed by consecutive NVT (at 300 K, 1000 ps) and NPT

dynamics (at 1 atm, 298 K, 500 ps) to ensure that a constant density has been reached. The equilibration steps for construction of PI microstructures are presented in **Table 1**. Two criteria were used to determine the equilibrium of the system: (1) The density of the system remained stable for a long time; (2) the fluctuation of energy was lower than 10 % [3]. The plots of density and energy versus simulation time in the last NPT for PI systems are shown in supporting information **Fig.S1** and **Fig.S2**. It can be seen that the PI systems have reached equilibrium states. The equilibrated densities of M-2,7-CPPI and 2,7-CPPI systems are 1.56 and 1.57 g/cm^3, respectively, which are in agreement with the experimental densities (1.56 and 1.57 g/cm^3). In all runs, Nosé method was used for temperature control [4]. In NPT runs, the pressure was controlled by Berendsen’s method. During these simulations, the cutoff for the nonbonded interactions was taken as 15.5 Å.

Table 1 The equilibration steps for construction of PI microstructures.

Steps	Simulation parameters	Total time
Construction of PI cell	300K	
System energy minimization	300K	
Annealing procedure	300K~1000K, steps of 50K, ten cycles, NPT	30 ns
Stage-wise equilibration procedure	1000K→600K, steps of 100K, NVT+ NPT<br>600K→400K, steps of 50K, NVT+ NPT<br>400K→300K, steps of 25K, NVT+ NPT	26 ns
Final relaxation	300K, NVT<br>298K, NPT	1 ns<br>0.5 ns

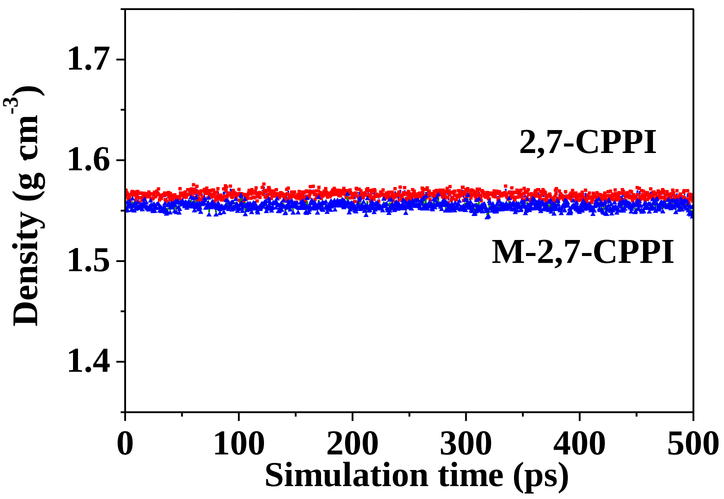

Fig. S1 Plots of density versus simulation time in the NPT simulation for M-2,7-CPPI and 2,7-CPPI.

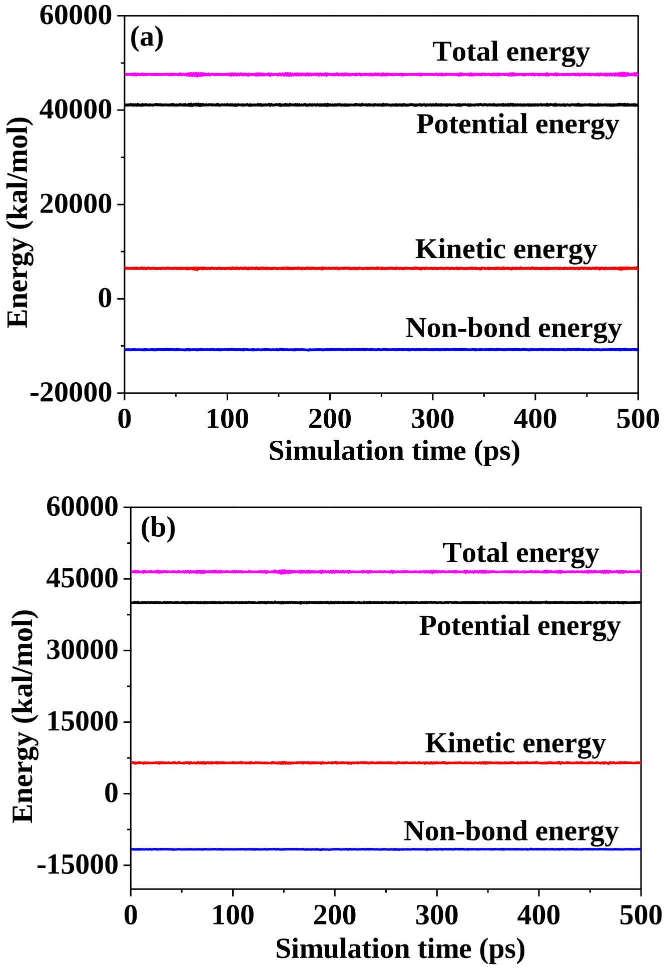

Fig. S2 Plots of energy versus simulation time in the NPT simulation for (a) 2,7-CPPI and (b) M-2,7-CPPI.

In order to prove the rationality of the PI models, the experimental and computed

glass transition temperatures (T_g) of M-2,7-CPPI and 2,7-CPPI are studied and shown in **Table 2**. The simulated specific volume as a function of temperature for M-2,7-CPPI and 2,7-CPPI are shown in **Fig.S3**. The T_g obtained from simulations of M-2,7-CPPI and 2,7-CPPI match to experimental values, with the errors lower than 10% (the errors between simulated/experimental values are shown in the brackets). These errors between simulated/experimental are within the margin error [5]. According to these data, these equilibrated cells can represent the structure of M-2,7-CPPI and 2,7-CPPI reasonably.

Table 2 Simulated and experimental T_g for M-2,7-CPPI and 2,7-CPPI.

**PIs**	**T_g (°C)**		
	**Exp(DMA)**	**Exp(DSC)**	**Sim**
2,7-CPPI	437	413	437(0%, 6%)
M-2,7-CPPI	437	395	428(2%, 8%)

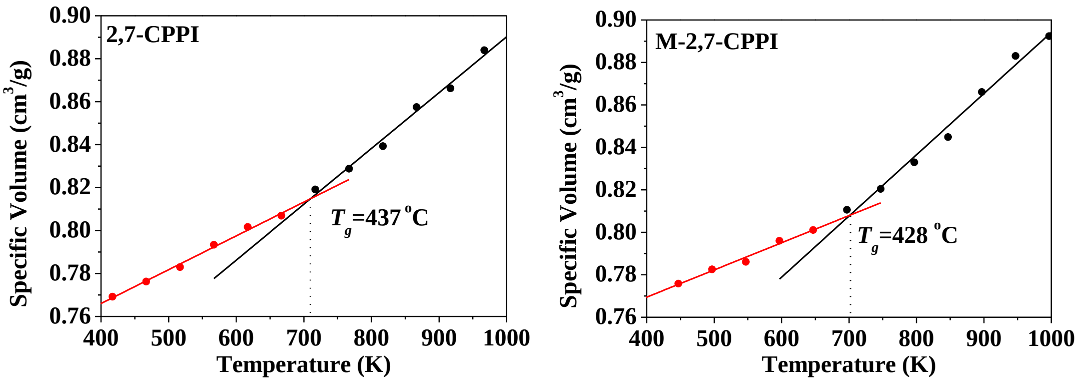

Fig. S3 The simulated specific volume as a function of temperature for M-2,7-CPPI and 2,7-CPPI.

2.2 Free volume

The free volume was determined by a grid scanning method using the Connolly task. The void distribution was estimated by a method previously used for micro-crystalline materials [6,7]. Specifically, the simulation cell was

divided into three-dimensional fine grids with a size of approximately 0.25 Å. The void size at a grid was determined as the diameter of the maximum cavity that encloses the grid and additionally has no overlap with any polymer atom.

2.3 Radius of gyration

In order to study the molecular conformation behavior of polymer chains, the radius of gyration (R_g) of polymer chains were calculated. R_g gives a sense of the size of the polymer coil and is defined as [8]:

$$R_g^2 = \frac{\sum_{i=1}^{N} |r_i - r_{cm}|^2 m_i}{\sum_{i=1}^{N} m_i} \qquad (1)$$

where r_i and r_{cm} represent the position vector of the *i*th atom and the center of mass of the polymer chain, respectively. m_i is the mass of site i and N is the number of atoms. An NPT simulation of the equilibrated system was performed for 300 ps to determine the R_g.

2.4 Radial distribution functions

The radial distribution functions (RDF) refer to a measure of the probability that, given the presence of an atom at the origin of an arbitrary reference frame, there will be an atom with its center located in a spherical shell of infinitesimal thickness at a distance r from the reference atom. The RDF was calculated by the average of the static relationship of every given pair of particles AB using the following equation [9]:

$$g_{AB}(r) = \frac{\langle n_{AB}(r) \rangle}{4\pi r^2 \Delta \rho_{AB}} \qquad (2)$$

where $\langle n_{AB}(r) \rangle$ is the average number of atom pairs between r and $r + \Delta r$, and

$\Delta\rho_{AB}$ is the density of atom pairs of type AB.

2.5 Diffusion coefficients

The diffusivity of gas molecules through PI was estimated by first inserting ten molecules of each gas into the equilibrated simulation box. Then, minimization of the potential energy was performed using ‘‘smart minimizing method’’ run. After this, the cell was put through annealing and stage-wise equilibration procedures using the same parameters as described before. The resulting structure was then equilibrated by NVT and NPT simulations at 298 K in order to ensure that its minimized total energy remained approximately constant with respect to the simulation time. An NVE simulation of the system was performed for 30000 ps. The diffusion coefficients can be calculated by means of the Einstein relation [10,11]

$$D = \frac{1}{6\mathrm{N}} \lim_{t\to\infty} \frac{\mathrm{d}}{\mathrm{dt}} \langle \sum_{i}^{N} |r_i(t) - r_i(0)|^2 \rangle \qquad (3)$$

where $\boldsymbol{N}$ is the number of penetrants, $\boldsymbol{r_i(0)}$ and $\boldsymbol{r_i(t)}$ are the initial and final positions of the center of mass of penetrant $\boldsymbol{i}$ over the time interval $\boldsymbol{t}$, and $\langle|\boldsymbol{r_i(t)} - \boldsymbol{r_i(0)}|^2\rangle$ is the averaged mean-square displacement (MSD) of the penetrant. The diffusion coefficient was determined from the slope of MSD versus time data. In this work, MSD of H_2O and O_2 were calculated from the trajectories of ten penetrant molecules in the PI microstructures.

2.6 Sorption isotherm

The equilibrated cell was used for grand canonical Monte Carlo (GCMC) simulations employing the standard Metropolis algorithm using the "Sorption Isotherm" module [12]. Both the polymer framework and the penetrant molecules were treated as rigid bodies. The degrees of freedom of the system were accordingly specified by the center-of-mass position and orientation of the molecules. Metropolis sampling was used for inserting or deleting permeant molecules as well as accepting or rejecting their translational and rotational configurational moves. The COMPASS force field and force field assigned partial charges on atoms were used. A VTμ simulation was performed at each fixed pressure and 298 K. The pressure of the penetrant gas was varied from 10 to 3000 kPa. For each pressure value, 10^5 equilibration steps were first performed to ensure proper relaxation of the polymer chains in response to the insertion of the penetrant molecule, following which 10^6 steps of production run were carried out. The sorption isotherm can be obtained in the form of a plot of the concentration of sorbed gas, C, as a function of pressure at constant temperature. The solubility coefficient, S, is then obtained from the limiting slope of the sorption isotherm at zero pressure as [13]

$$S = \lim_{p \to 0} (C/p) \qquad (4)$$

where C is in units of cm^3(STP)/cm^3(polymer) and p is pressure.

3. Characterization and properties of monomers and polyimides

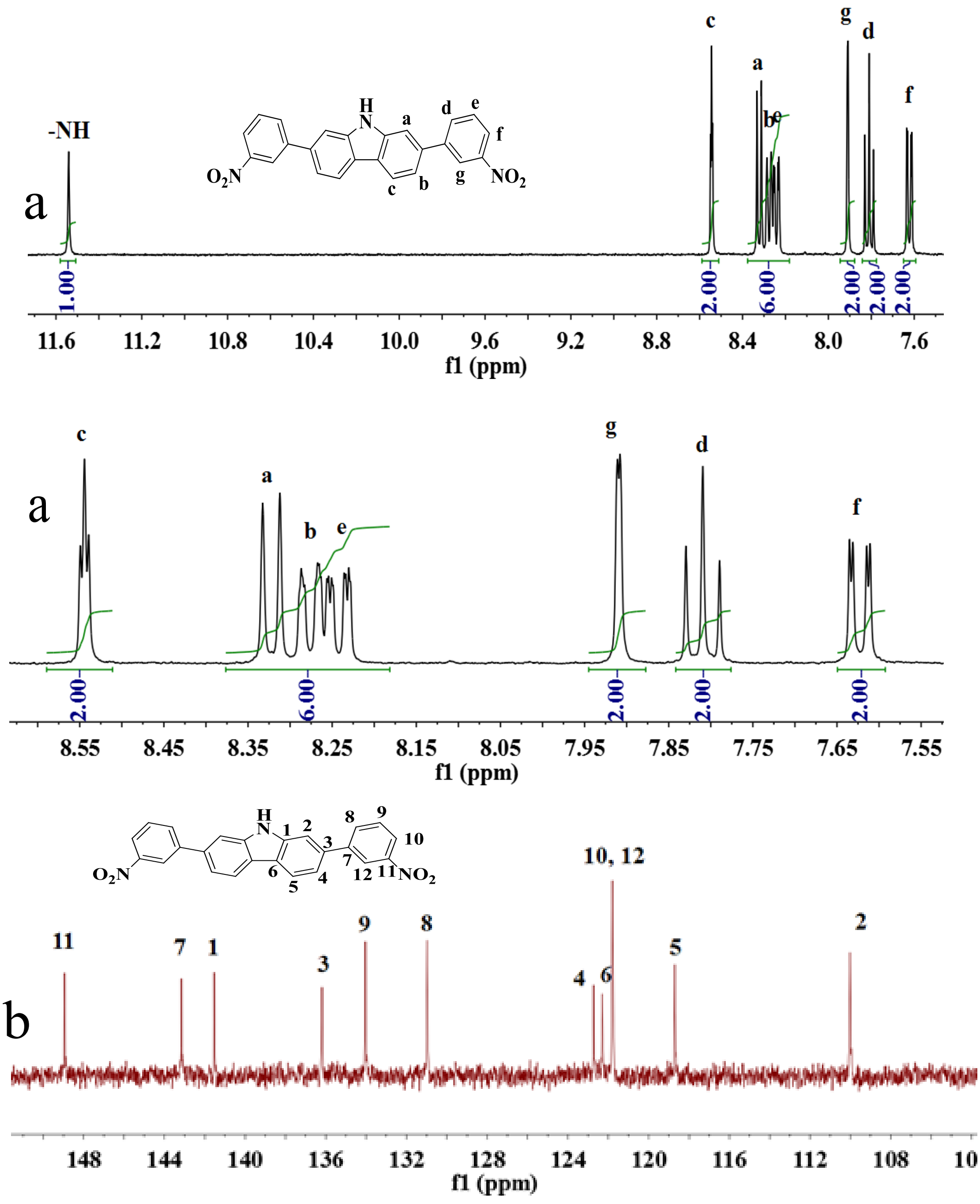

Fig. S4 ^{1}H NMR (a) and ^{13}C NMR (b) spectra of M-2,7-CPDN.

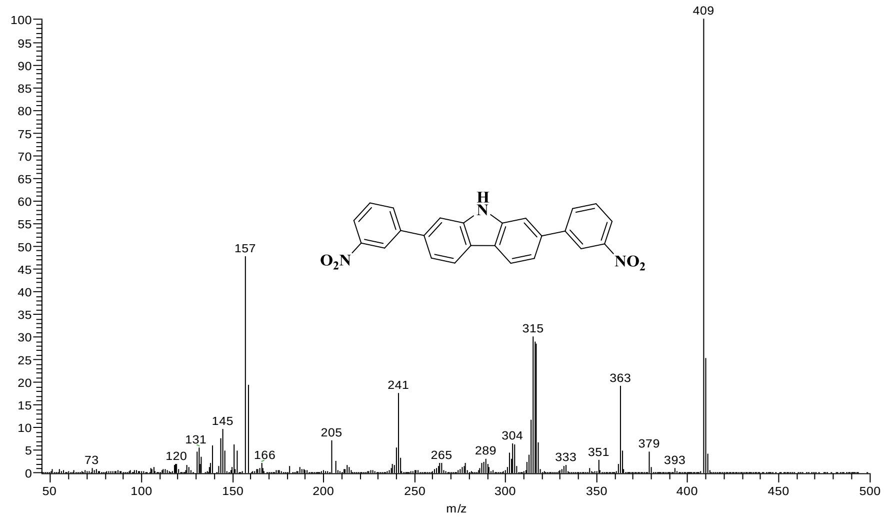

Fig. S5 Mass spectrum of M-2,7-CPDN

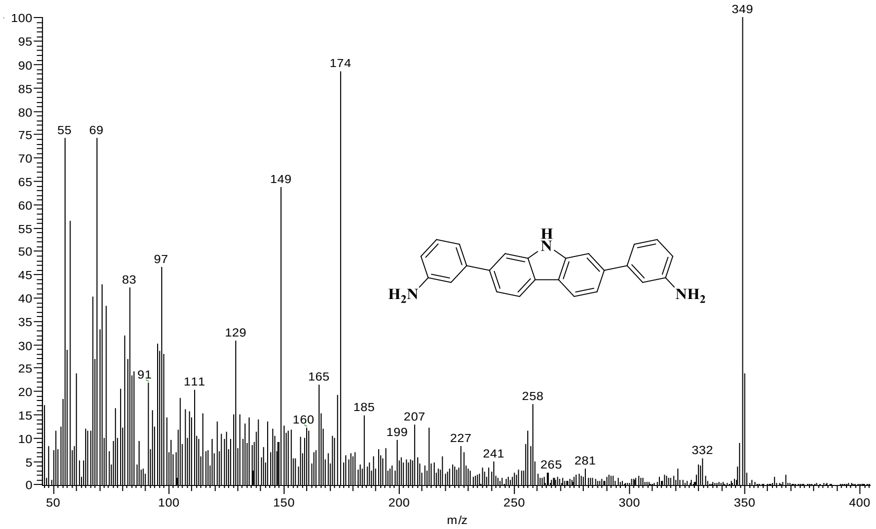

Fig. S6 Mass spectrum of M-2,7-CPDA

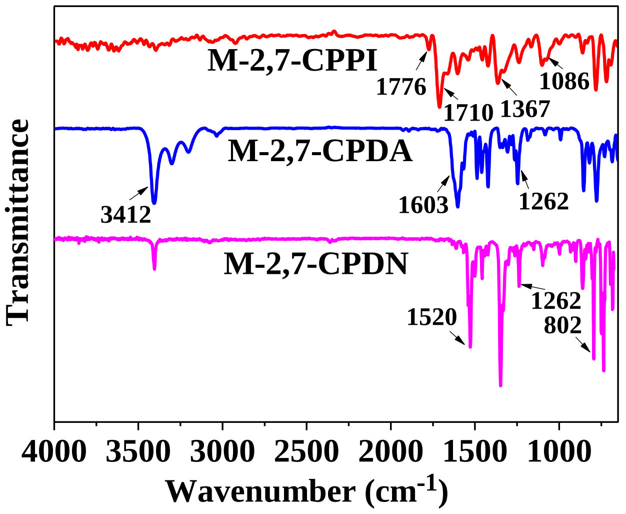

Fig S7 FT-IR spectra of M-2,7-CPDN, M-2,7-CPDA and M-2,7-CPPI

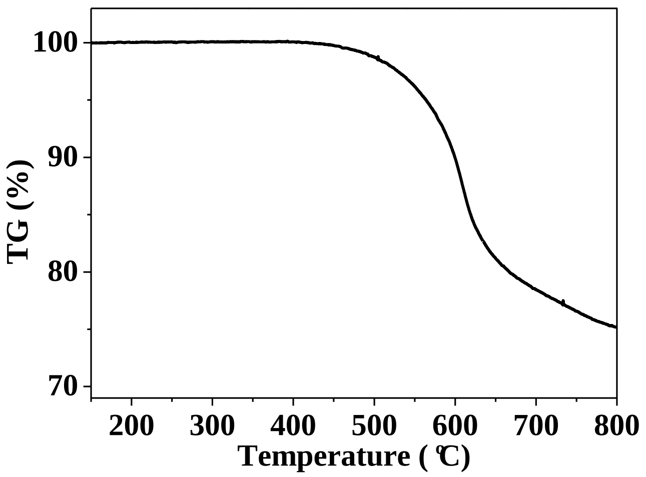

Fig. S8 TGA curve of the M-2,7-CPPI film

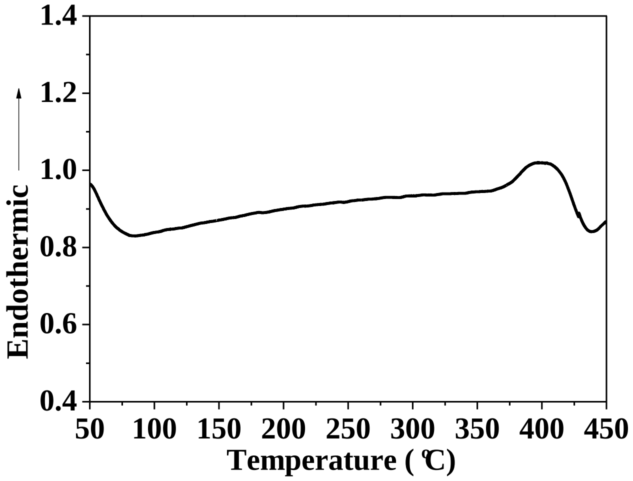

Fig. S9 DSC curves of the M-2,7-CPPI film

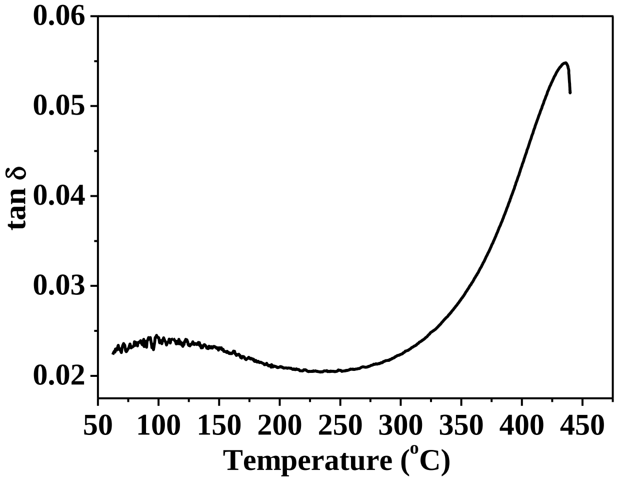

Fig. S10 DMA curve of the M-2,7-CPPI film

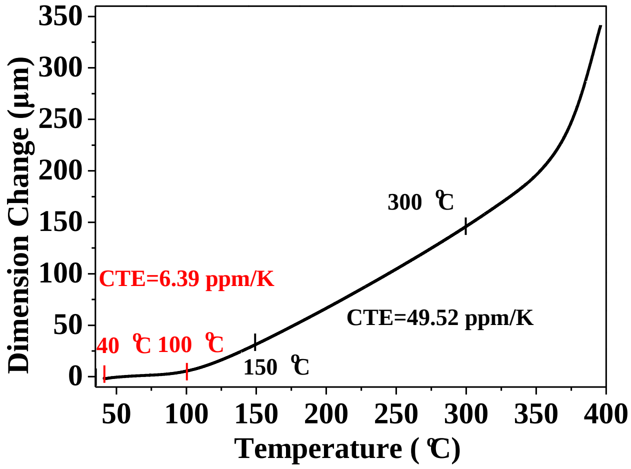

Fig. S11 TMA curve of the M-2,7-CPPI film

4. Aggregation structures analysis by radial distribution functions

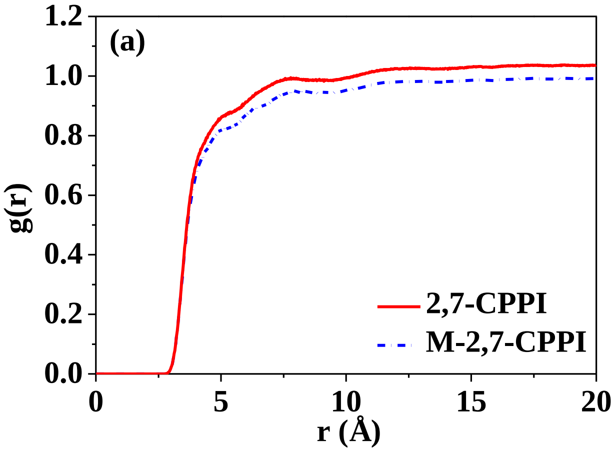

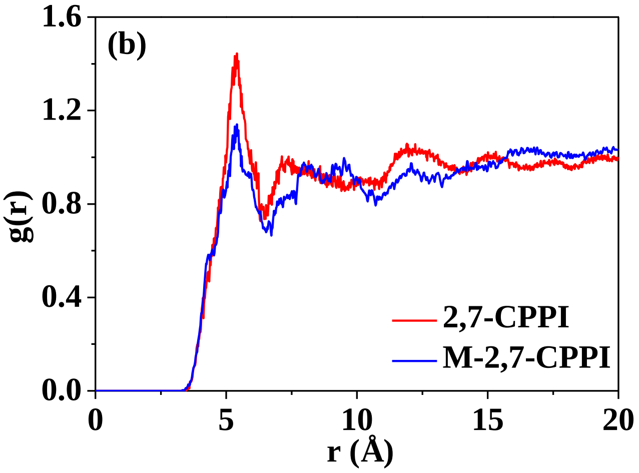

Fig. S12 Chain-chain intermolecular RDFs based on (a) all the carbon atoms in the benzene rings and (b) all the nitrogen atoms in the imide rings for 2,7-CPPI and M-2,7-CPPI.

5. Radius of gyration analysis

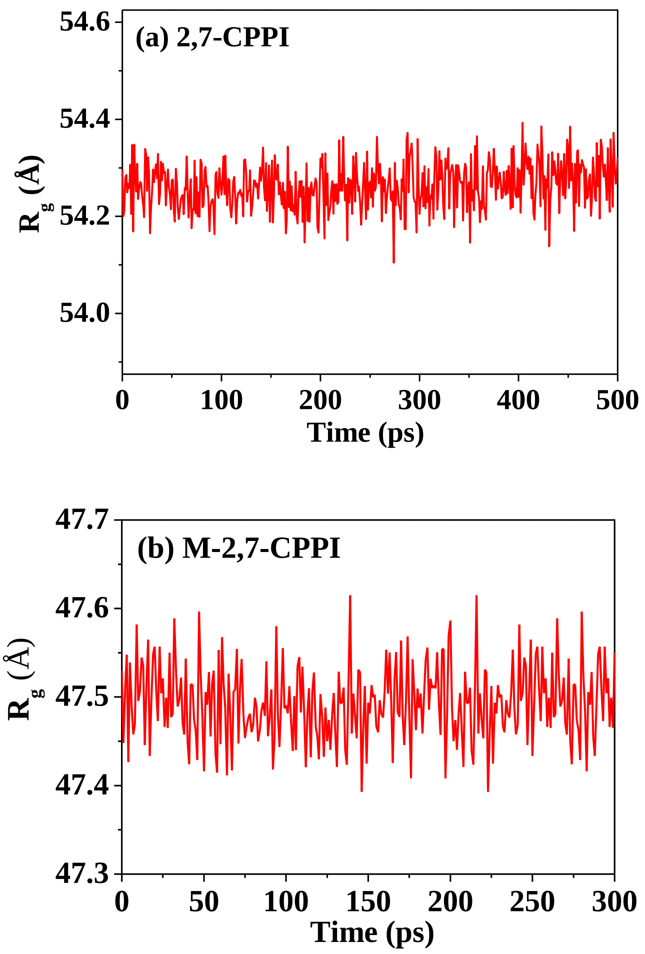

Fig. S13 Time dependence of the radius of gyration R_g for (a) 2,7-CPPI and (b) M-2,7-CPPI.

6. Gas diffusion

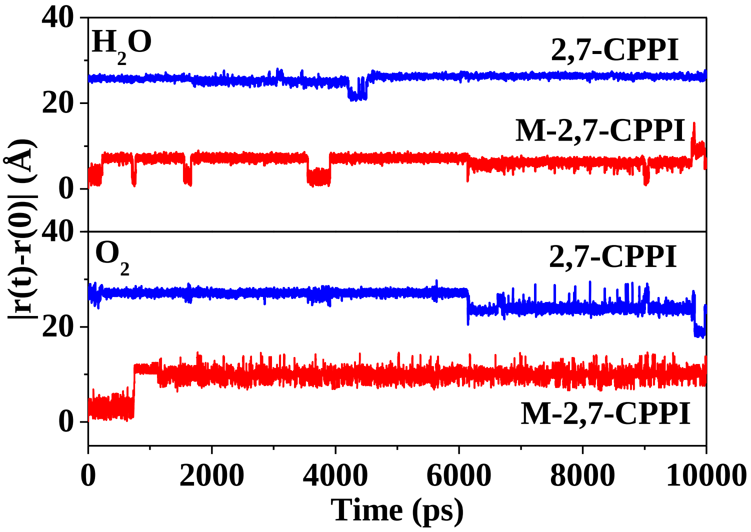

Fig. S14 Displacement of O_2 and H_2O from their initial positions in M-2,7-CPPI and 2,7-CPPI. The curves of O_2 and H_2O in 2,7-CPPI have been shifted vertically by 25 Å for better visualization.

References

1. Sun, H. COMPASS: An ab initio force-field optimized for condensed-phase applications -Overview with details on alkane and benzene compounds. *J. Phys. Chem. B* **1998**, *102*, 7338-7364.
2. Michael, J. M.; Sun, H.; Rigby, D. Development and validation of COMPASS force field parameters for molecules with aliphatic azide chains. *J. Comput. Chem.* **2004**, *25*, 61-71.
3. Zhu, J.; Zhao, X.; Liu, L.; Song, M.; Wu, S. Quantitative relationships between intermolecular interaction and damping parameters of irganox-1035/NBR hybrids: A combination of experiments, molecular dynamics simulations, and linear regression analyses. *J. Appl. Polym. Sci.* **2018**, *135*, 46202.
4. Nosé, S. A unified formulation of the constant temperature molecular dynamics methods. *J. Chem. Phys.* **1984**, *81*, 511-519.
5. Kucukpinar, E.; Doruker, P. Molecular simulations of gas transport in nitrile rubber and styrene butadiene rubber. *Polymer* **2006**, *47*, 7835-7845.
6. Ban, S.; Vlugt, T.J.H. Zeolite microporosity studied by molecular simulation. *Mol. Simul.* **2009**, *35*, 1105-1115.
7. Bhattacharya, S.; Gubbins, K.E. Fast method for computing pore size distributions of model materials. *Langmuir* **2006**, *22*, 7726-7731.
8. de Oliveira, O.V.; Costa, L.T.; Leite, E.R. Molecular modeling of a polymer nanocomposite model in water and chloroform solvents. *Comput. Theor. Chem.* **2016**, *1092*, 52-56.
9. Zeng, J.; Zhang, Y.; Dai, Y.; Chen, S. Molecular dynamics simulation of nitrobenzene in heterocyclic ionic liquids. *J. Mol. Liq.* **2014**, *198*, 274-279.
10. Pant, P.V.K.; Boyd, R.H. Molecular dynamics simulation of diffusion of small penetrants in polymers. *Macromolecules* **1993**, *26*, 679-686.
11. Mullerplathe, F. Molecular dynamics simulation of gas transport in amorphous polypropylene. *J. Chem. Phys.* **1992**, *96*, 3200-3205.
12. Akkermans, R.L.C.; Spenley, N.A.; Robertson, S.H. Monte Carlo methods in Materials Studio. *Mol. Simul.* **2013**, *39*, 1153-1164.
13. Fried, J.R.; Sadat-Akhavi, M.; Mark, J.E. Molecular simulation of gas

permeability: poly(2,6-dimethyl-1,4-phenylene oxide). *J. Membr. Sci.* **1998**, *149*, 115-126.
